# Is the glass half full or half empty?

**DOI:** 10.2349/biij.3.2.e48

**Published:** 2007-04-01

**Authors:** BJJ Abdullah, KH Ng

**Affiliations:** Department of Biomedical Imaging, Faculty of Medicine, University of Malaya, Kuala Lumpur, Malaysia

“To seek to dispose of a major scientific issue by a show of hands is a striking demonstration of the way in which belief can come to dominate the thinking of scholars.” - Derek Freeman

Numerous new ideas, concepts, products and services are constantly being introduced from a wide variety of sources. These sources range from trend-setting teens and twenty-somethings in the nation’s metropolitan centres to established corporations offering new products for better health, living and longevity, as well as better diagnosis and treatment. This is the nature of modern culture. Some of these achieve a measure of consistent success, some fail, and some take off on an upward trajectory of exponential popularity and influence until they, too, get replaced by the next wave of “newness”. Why, one wonders, do ideas, concepts and values change with time? What drives some to an exalted place in world affairs while other seemingly better ones are relegated to the sidelines hoping for a day in the limelight?

Several different approaches, ranging from “memes” [1] to “tipping point” to Kuhn’s paradigms [2], have been promoted to explain how concepts, ideas or values change. Richard Dawkins’ ‘meme’ (rhyming with ‘gem’) refers to a “unit of cultural information” which can propagate from one mind to another in a manner analogous to genes (i.e., the units of genetic information). These include things such as tunes, catch-phrases, beliefs, fashions, ways of making pots, scientific and medical theories or ways of building arches. In reality, memes frequently propagate not as single entities but rather as integrated cooperative sets or groups (memeplexes or meme-complexes). The concept of ‘memes’ in itself is a successful meme which is now accepted in popular culture. Interestingly there are those who propose that memes evolve via natural selection through variation, mutation, competition, and ‘inheritance‘ of influences to replicate their success akin to Charles Darwin’s concept of biological evolution. This means that it is the modification of the original concept/idea that allows some ideas to survive, spread, and mutate while those that do not undergo such changes or are resistant to staying relevant with the times face oblivion. Evolution of memes has to be an active process where the brain creates and modifies them all the time. We may all be listening to or reading the same things but our brains are actively modifying or interpretating them into very different forms.

A paradigm, in Kuhn’s view, originates from the ‘great works’ of science, like Copernicus’s De Revolutionibus or Newton’s Principia because they were “sufficiently unprecedented to attract an enduring group of adherents away from competing modes of scientific activity,” and “sufficiently open-ended to leave all sorts of problems for the redefined group of practitioners to resolve” [2].

Lest we run away with the idea that having memes, ideas, concepts/paradigms is totally bad, handicaps progress and stifles new thinking, we should acknowledge that there is certainly a role for them. People study these paradigms in order to become members of the particular community in which they will later practice. In these communities, the student largely learns from, and is mentored by, teachers who learned the basics of their field from the same concrete models. Thus there is seldom disagreement over fundamentals and all eventually become committed to the same rules and standards for scientific practice. This sharing of a common paradigm ensures that its practitioners engage in observations that fit into their own paradigm i.e., investigate the kinds of research questions to which their own theories can most easily provide answers. Therefore paradigms help scientific communities to form boundaries around their discipline, in that they help the scientist to create avenues of inquiry, formulate questions, select methods with which to examine questions, define areas of relevance and possibly establish/create meaning [2]. In the absence of a paradigm or some candidate for a paradigm, all the facts that could possibly pertain to the development of a given science are likely to seem equally relevant. Therefore paradigms are essential to scientific inquiry as no natural history can be interpreted in the absence of at least some implicit body of intertwined theoretical and methodological belief that permits selection, evaluation, and criticism [2].

It must be stressed that modifications of concepts/ideas are not always about trying to be better; they are sometimes purely about survival, even if the consequences are dire. Man has evolved to live with these negative effects when they occur. Just like the evolution of genes where multiple factors, not just the success of the species as a whole, influence the evolution of genes, the evolutionary pressures on memes include much more than just truth and economic success.

Evolutionary pressures on memes, ideas, concepts/paradigms may include the following:

- Experience: This is similar to the concept of ‘power of context‘ in the tipping point approach [3]. Experience will probably include beliefs, values and world views within which each of us operate. This is enormously important in determining whether a particular phenomenon will tip into widespread popularity. Even minute changes in the environment, e.g. small variations in social groups and minor changes in a neighborhood or community environment can play a major factor in the propensity of a given concept attaining the tipping point. If a meme does not correlate with an individual’s experience or his world view, then this individual is less likely to remember that meme or incorporate it into current concepts. In fact, the more the idea challenges his current views, the less likely he is to even give the idea a second thought before throwing it out the window. When such discomfort occurs, one needs to recognise it as a challenge, as being a new point of view. One should not withdraw from it but rather force oneself to look at it as objectively as possible. What may be even more important is the use of these experiences, beliefs and values to fill up gaps in our understanding/knowledge to allow us to continue functioning. Otherwise we would be left in a state of uncomfortable limbo. Scientists, of course, also hold beliefs that go beyond the scientific evidence. To what extent, it is fair to ask, are the interpretations given to scientific evidence shaped by the world view of the scientist? [4]. A scientific community cannot practise its trade without some set of received beliefs. These beliefs form the foundation of the “educational initiation that prepares and licenses the student for professional practice” and the ‘rigorous and rigid‘ nature of the preparation helps ensure that the received beliefs exert a ‘deep hold‘ on the student’s mind [2].
- Pleasure/Pain/Rewards: If a meme results in rewards that the individual desires, be it more pleasure or less pain, monetary or personal gain, then there is increased likelihood of acceptance. However, if the road forward requires sacrifices, loss of position and stature or additional work, then the rewards must truly be great, for most would discard the idea! One could even go so far as to say that support can be bought for the right price, e.g. a promotion or an award. We are all very familiar with the future rewards, be it here on earth or hereafter.New assumptions (paradigms/theories) require the reconstruction of prior assumptions/concepts/theories and the reevaluation of prior facts. This is difficult and painful for the individual and community, plus it is time consuming and is also strongly resisted by the established community. Most of us find it hard to accept little changes like how the toothpaste tastes, let alone more fundamental changes in the way people view the world, its relationships and workings. Changes in such paradigms are therefore exhausting. These scientific revolutions occur when an anomaly subverts the existing tradition of scientific practice. However, on the bright side, when such a shift does take place, the scientist’s world is qualitatively transformed and quantitatively enriched by fundamental novelties of either this new fact or theory.
- Fear/Bribery/Punishment: It is well recognised that the incumbents, by virtue of their position, have a great advantage over any challenger and their ideas. This is because the ‘believers‘ are in a position to punish or withhold recognition from those who deviate from conventional wisdom or have the gall to challenge the status quo. The incumbents also have powerful tools at their disposal; they are able to create edicts and laws, terrifying scenarios of the future and even use force. The challengers may be called heretics, madmen, trouble makers, the devil’s workmen or just plain jealous people. Copernicus was made to drink poison for saying that the earth rotated around the sun. In this way, non-believers may be frightened into remaining as believers, at least on the surface. This tool has probably been most widely exploited over the centuries to keep non-believers in line. It has also enabled the survival of philosophies, ideas, beliefs and concepts despite all the changes occurring around us.
- Censorship: If a group of individuals or an organisation controls the usage or the dissemination of a meme, then the success of any competing concept/idea/meme may suffer a selective disadvantage. This may occur by rejecting publication of the “offensive” material so it is not able to have acceptance in the community. This thus prevents it from being published in the top peer-reviewed journals. And since there is minimal or no scientific evidence in the literature to support the meme, it is not ‘scientifically‘ proven or accepted. In other extreme circumstances, leaders have tried to destroy any retention-systems containing a particular meme by destroying the books or libraries containing these materials. This then allows them to establish their own memes, be they religious, political or even social. It has even been said that normal science often suppresses fundamental novelties because they are necessarily subversive of its basic commitments [2]. However, with the advent of the internet, which has given rise to email, online working groups, blogs, electronic journals, etc, the role of censorship has decreased tremendously, since other means of dissemination are both ineffective and costly. This obviously raises the question of the validity of the information being disseminated.
- Economics: If people or organisations with economic influence exhibit a particular meme, then the meme has a greater likelihood of benefiting from a greater audience. If a meme tends to increase the riches of an individual holding it, then that meme may spread because of imitation. Such memes might include ‘Hard work is good‘ and ‘Put number one first‘.
- Distinction: This is usually the most important factor that decides the fate of a new idea or concept. Before there is widespread acceptance of an idea, a few key types of people (leaders, intelligent people, celebrities, sports personalities, insightful people, recognised publications, respected organisations, etc) must champion an idea, concept, or product. If the meme enables hearers to recognise and respect the tellers, then the meme has a greater chance of attaining widespread popularity. The ‘bigger‘ the person or organisation, the greater the credibility associated with them. By converting to this new view, or an evolved/mutated version, this ‘superior‘ knowledge can provide a promotion to elite status. This is commonly seen in organisations, from businesses to political parties, where change in leadership results in a change in the ‘speak‘. Those who wish to continue being in the elite group demonstrate a change in priorities, values and orientation towards the new leader.
- Other factors which have been said to influence the success or failure of an idea is the Stickiness Factor (a unique quality that compels the phenomenon to ‘stick‘ in the minds of the public and influence their future behavior) [3]. Often, the way that the Stickiness Factor is generated is unconventional, unexpected, and contrary to received wisdom. Another possible way of looking at this is the advent of popular science or medicine written for the lay public in ways that they understand and appreciate, for example with the use of catchy headlines like ‘Walking away from paralysis‘.

In our recorded history, numerous philosophies or paradigms have evolved and developed to benefit the societies that embrace it. A legacy of some modern philosophers in science and philosophy was to assemble memetic systems that continuously question paradigms whenever additional information becomes available. When gaps or conflicts occur in these paradigms, scientists and philosophers may either seek a theoretical or empirical solution to resolve them. The theoretical solution would involve mathematical analyses, thought experiments, logic or analysis while the empirical solutions would either be experimental or observational studies.

One of the key factors that laid the foundation for science, medicine, and philosophy was the ethical, moral, and scientific obligation to not accept anything at face value. It required one to consistently and persistently question all that is being put forth. The consequence is that nothing is accepted as true unless empirical evidence and observation suggests such ‘truth‘ strongly and consistently. The great thinkers of our time who pushed these frontiers included Socrates, Aristotle, Plato, Copernicus, Newton, Darwin, Albert Einstein and Karl Marx. We often forget that what is today’s norm, practice and accepted knowledge had first to be argued by a lone voice dissenting against huge established incumbent resistance, with the intentional use (or otherwise) of the evolutionary pressures. These lone voices are often labeled as heretics, trouble makers and even conspirators.

Despite all the proven benefits of ionising radiation, from use in agriculture to pest control, from energy generation to diagnosis and treatment, from manufacturing to space travel, we are constantly being reminded that the exposures should be kept in line with the concept of ALARA. The general radiation safety policy, agreed in consensus by the International Commission on Radiological Protection (ICRP), the United Nations Scientific Committee on the Effects of Atomic Radiation (UNSCEAR), the UK’s Radiation Protection Division of the Health Protection Agency (formerly the National Radiological Protection Board, NRPB) and the National Council on Radiation Protection and Measurement (NCRP) in the USA, is based on the assumption that the risk of a radiation-induced fatal cancer is linearly proportional to the dose and therefore every effort should be made to keep exposure to the minimum. What this means in assessing the risks versus benefits of low-level radiation exposure is that even very, very low levels of exposure to ionising radiation carry an associated risk, albeit a small one, of developing cancer as a result of this exposure. This is known as the linear, no-threshold (LNT) model of radiation risk. This LNT model should not be regarded as immutable law, proven in every circumstance, but rather as a robust working rule [5].

In contrast to this, there are those who propose that low-dose radiation may actually stimulate the immune system. Radiation hormesis is the theory that ionising radiation is benign at low levels of exposure, and that doses at the level of natural background radiation can be beneficial. This concept proposes that there is such a thing as ‘radiation deficiency‘ where people living in areas with much lower background radiation levels may suffer higher cancer death rates [6]. This is an extension of the concept of hormesis which has been around since the 1980s [7,8].

Radiation hormesis has been rejected by both the United States National Research Council (part of the National Academy of Sciences) [1] and the National Council on Radiation Protection and Measurements (a body commissioned by the U.S. Congress) [2]. In addition, the United Nations Scientific Committee on the Effects of Ionizing Radiation (UNSCEAR) wrote in its most recent report [3]:

Until the [...] uncertainties on low-dose response are resolved, the Committee believes that an increase in the risk of tumour induction proportionate to the radiation dose is consistent with developing knowledge and that it remains, accordingly, the most scientifically defensible approximation of low-dose response. However, a strictly linear dose response should not be expected in all circumstances.

Numerous studies suggest the possibility that low doses of radiation may be benign. The disagreement arises partly because very low doses of radiation have relatively small impacts on individual health outcomes and therefore, it is difficult to detect the ‘signal’ of decreased or increased morbidity and mortality due to low-level radiation exposure amidst the ‘noise’ of other effects. Some of the questions raised from the studies in favour of radiation hormesis are:

- Free radicals created in the normal process of metabolism, resulting from routine eating and breathing and the stress of heat and exercise (approximately a million DNA nucleotides in each cell damaged each day), cause the most damage [9] including double DNA breaks [10] compared to ionising radiation. When one looks at even higher levels of radiation, only a few more mutations are added to those millions occurring from metabolism [11].
- The ratio of the probabilities for radiation-induced lethal cancer and the corresponding DSB is about 10-11 to 10-12, e.g. from 100 kVp X-rays, on potentially oncogenic stem cells with an average mass of 1 nanogram [12-14].
- Studies over the past 25 years raise the possibility of an adaptive protection response occurring in mammalian cells in vivo and in vitro after single as well as protracted exposures to X- or c-radiation at low doses [15].
- Animal studies have shown that radiation exposure enhances the biological response of immune systems [16-18] with no evidence of chromosomal damage for several generations [19-22].
- Based on some studies [23, 24], there is no human data to support this assumption of LNT for a short-term dose below 0.2 Gy (centi-grays), i.e. the equivalent of about two centuries of exposure to natural gamma radiation [25-27]. The debate over these results rages on [28].
- Contrary to conventional wisdom, studies have raised the possibility that the higher the radon levels, the lower the incidence of lung cancer [29-33].
- Similar questions have been raised with regard to incidence of bone sarcomas in radium dial painters, from studies of radium cases in 1970-1980s [34-37].
- Are the benefits of mammography the result of the screening or the result of the radiation exposure? There has been data from a Canadian study [38, 39] looking at breast cancer in pulmonary TB patients who had chest fluoroscopy as part of their management, which once again raises challenging questions. What this study reports is that below a dose of approximately 30 cGy (centi-grays), there was a highly statistically significant reduction in breast cancer [40]. Could one examining the available data on mammography get similar results based on the assumption that it was the low level radiation exposure that led to these outcomes?
- If one then examines data on treatment of some cancers using low-dose radiation, one gets even more astonished/discouraged/confused [41-45].
- A fact unknown to most people is that there are higher background radiation doses in health spas where people go to rejuvenate themselves! [7, 8]

The direct consequence of this theory is that it challenges the linear non-threshold theory. If there is a threshold, billions of tax dollars worldwide can be saved annually from unnecessary measures. The industry of radiation protection, as well as all the rules and regulations related to it would have to be re-examined while the cost inherent in reducing the exposure of radiation workers and the public would decrease dramatically. Would this spawn a whole new industry for the use of radiation therapy in disease prevention and health maintenance? This would definitely include the use of total body screening using CT.

Officially, the jury may still be out, but in this age of evidence based medicine, more evidence needs to be generated to substantiate or refute this very interesting and controversial view of low-dose exposure and increased longevity. But in the meantime, it is the special responsibility of scientists to inform the world of the choices. The question of the benefits of low level radiation has been raised not by crazy, irrelevant riff raff but by numerous experts in their field, which certainly raises the seriousness of the issue. The discussions are often so complex and involve such complicated statistical analyses and methods that even professionals within the same specialty find themselves truly lost.

One then wonders that if scientists all claim to believe in the scientific method, and if they all have access to the same data, how can there be such deep disagreements among them? What separates the two sides in most scientific controversies, however, is not so much an argument over the scientific facts, scientific laws or even the scientific method. It is, instead, an argument about values [46].

You may ask why this is so. Firstly there are huge gaps in the data leading to our understanding of the complex integrated biological and physical systems. As occurs in most specialist debates, the use of numbers, complex statistics and equations makes the combatants sincerely believe that they are engaged in a purely scientific, impartial and objective debate. However, most scientists come into the profession with their own personal world views, be they political, social, religious, or cultural, long before they were exposed to science in a serious way [46]. When faced with gaps in their knowledge or understanding of related issues, the tendency is to fill the gaps with the ’unscientific‘ value-based perspectives. This in itself is not bad, since we all need to operate within the wider cultural, political and social context, but what is dangerous is that this ‘value based’ perspective is not recognised. Has history not recorded enough pain, suffering and loss brought about by dogmatic views in medicine, science, politics and religion?

It is undeniable that scientists are influenced by their beliefs [46]. But so long as both sides adhere to the scientific process and do not resort to emotional approaches, name calling and other under-handed techniques, these differences in position are very powerful motivations for better science. In any debate, each side knows that every flaw in their data, or oversight in their analysis, will be seized upon by their opponents. Both sides will strive to produce better data and better analysis in the conviction (faith, if you wish) that the truth will vindicate their prejudice. The numbers, when science finally learns them, will ultimately decide the winner. In the end, the result will be a better understanding of the global climate. To the frustration of its postmodern critics, science works [46].

But it will be many years before the true understanding of the effects of low-dose radiation are known for us to make clear choices. There are those who believe that it may be premature for us to change our practice until we have the necessary information, since the consequences to radiation workers, the public and future generations may be tragic. Then there are those who feel that waiting until sufficient data is collected may entail too much cost for unnecessary procedures, and raise unreasonable fear about radiation; these people question if the data will ever be enough for a change in the status quo.

We should try to be open and explore these questions so that the truth can be uncovered. To quote Geoff Watts, “Knowledge doesn’t suddenly appear in neat and tidy quanta. Like patches of lichen spreading over a rock face, it accretes over decades.” [47]. Science works precisely because its results are always tentative. When newer and better information becomes available in medicine and science, entire textbooks are rewritten with hardly a backward glance. Unfortunately many people, both within and outside the business, are uneasy standing on such loose soil; they seek a certainty that science cannot offer [46].

As mentioned, the prevailing mindset and ascendancy of one viewpoint may be detrimental to the long-term interest of the people we serve to protect. It may be time for the issue of low-dose radiation to be explored, not just by radiation protection-oriented researchers but by specific disciplines, for example, immunology, genetics, and so on. The discussions must be put into plain and simple language so that those who are not truly experts may be party to the discussions and have their viewpoints shared. It is not uncommon to read comments by professionals who pretend to understand this complex issue but are really out of their depth.

In order to properly assess low-dose effects, all studies should analyse the dose range below the level at which adverse effects are demonstrated. Data from research studies which raise these questions and go against conventional wisdom have not been published, one wonders why? In addition, independent assessment of the data for rule-making by government agencies must incorporate the scientists and analysts who have documented for decades that radiation health effects data cannot be linear. Until the controversy is resolved, physicians must minimise radiation exposure by following the “do not harm” and “as low as reasonably achievable” principle.

We have viewed the discoveries of X-ray and radioactivity as blessings for mankind but have been made acutely aware of the hazards and the need for radiation protection. Maybe there is more benefit to radiation exposure than we have thought or accepted possible. It may be that all that is set out by the ’new‘ may not come to bear, but also all that is held to be gospel with regard to NLT may also be holding an unreasonable position.

But until then, we should not be arguing if the glass is half full OR half empty but rather agree that it is BOTH half empty and half full and ask ourselves what we can do with what’s in the glass openly and honestly.

## References

[1] Dawkins R (1990). The Selfish Gene.

[2] Kuhn T (1996). The Structure of Scientific Revolutions.

[3] Gladwell M (2000). The Tipping Point: How Little Things Can Make A Big Difference.

[4] Ashman K e (2000). After the Science Wars: Science and the Study of Science.

[5] Wall BF, Kendall GM, Edwards AA (2006). What are the risks from medical X-rays and other low dose radiation?. Br J Radiol.

[6] Jagger J (1998). Natural background radiation and cancer death in Rocky Mountain states and Gulf Coast states. Health Phys.

[7] Luckey T D (1980). Hormesis with Ionizing Radiation.

[8] Luckey T D (1991). Radiation Hormesis.

[9] Bishop M (1989). Cancer.

[10] Billen D (1990). Spontaneous DNA damage and its significance for the "negligible dose" controversy in radiation protection. Radiat Res.

[11] Varmus H, Weinberg R A (1993). Genes and the Biology of Cancer.

[12] Feinendegen LE, Loken MK, Booz J (1995). Cellular mechanisms of protection and repair induced by radiation exposure and their consequences for cell system responses. Stem Cells.

[13] Feinendegen L E, Bond V P, Sondhaus C A (2000). The dual response to low-dose irradiation: Induction vs. prevention of DNA damage.

[14] Feinendegen LE (2003). Relative implications of protective responses versus damage induction at low dose and low-dose-rate exposures, using the microdose approach. Radiat Prot Dosimetry.

[15] UNSCEAR (1994). Sources and Effects of Ionizing Radiation, Annex B, Adaptive Responses to Radiation in Cells and Organisms.

[17] Walinder G (1995). Has Radiation Protection Become a Health Hazard?.

[18] Lorenz E (1954). Biological Effects of External Gamma Radiation, Part I.

[19] LORENZ E (1950). Some biologic effects of long continued irradiation. Am J Roentgenol Radium Ther Nucl Med.

[20] Brucer M (1990). A Chronology of Nuclear Medicine.

[21] HENRY HF (1961). Is all nuclear radiation harmful?. JAMA.

[22] Feinendegen L E, Bond V P, Sondhaus C A (1998). Can Low Level Radiation Protect Against Cancer?. Physics and Society.

[23] Matanoski G (1991). Health effects of low-level radiation in shipyard workers, Final report.

[24] Luckey T D (1997). Ionizing Radiation Decreases Human Cancer Mortality Rates. Low Doses of Ionizing Radiation, Biological Effects and Regulatory Control.

[25] Cameron JR (1992). The Good News About Low Level Radiation Exposure. Health Physics Society Newsletter.

[26] Cameron JR (2001). Is Radiation an Essential Trace Energy?. The American Physical Society.

[27] Smith PG, Doll R (1981). Mortality from cancer and all causes among British radiologists. Br J Radiol.

[28] Wagner LK (2003). The "Healthy Worker Effect": science or prejudice?. Radiology.

[29] Cohen BL (1987). Tests of the linear, no-threshold dose-response relationship for high-LET radiation. Health Phys.

[30] Cohen BL (1989). Expected indoor 222Rn levels in counties with very high and very low lung cancer rates. Health Phys.

[31] Cohen BL (1995). Test of the linear-no threshold theory of radiation carcinogenesis for inhaled radon decay products. Health Phys.

[32] Cohen BL, Colditz GA (1994). Tests of the linear-no threshold theory for lung cancer induced by exposure to radon. Environ Res.

[33] Bogen K (1996). A Cytodynamic Two-Stage Model That Predicts Radon Hormesis (Decreased, then Increased Lung-Cancer Risk vs. Exposure).

[34] Evans R D (1983). Highlights of the Meeting - Invited Summary. In:Radiobiology of Radium and the Actinides In Man, Proc of an Int’l Conf.. Health Phys.

[35] Thomas R (1994). The U.S. Radium Luminisers: A Case for a Policy of ‘Below Regulatory Concern’. J Radiol Prot.

[36] Maletskos C J (1994). Radium in Man-20 Years Later. ANS Transactions.

[37] Rowland R (1997). Bone Sarcoma in Humans Induced by Radium: A Threshold Response?.

[38] Muckerheide J e (1998). Radiation, Science, & Health. Low-Level Radiation Health Effects: Compiling the Data.

[39] Muckerheide J (1995). The Health Effects of Low Level Radiation: Science, Data, and Corrective Actions. Nuclear News.

[40] Makinodan T (1992). Cellular and Subcellular Alterations in Immune Cells Induced by Chronic, Intermittent Exposure in Vivo to Very Low Doses of Ionizing Radiation and Its Ameliorating Effects on Progression of Autoimmune Disease and Mammary Tumor Growth.

[41] Hattori S (1997). State of Research and Perspective on Adaptive Response to Low Doses of Ionizing Radiation in Japan. IAEA.

[42] Sakamoto K, Miyamoto M, Watabe N (1987). [The effect of low-dose total body irradiation on tumor control]. Gan To Kagaku Ryoho.

[43] Sakamoto K (1996). Fundamental and Clinical Studies on Tumor Control. ANS Transactions.

[44] Miyamoto M, Sakamoto K (1987). [Anti-tumor effect of total body irradiation of low doses on WHT/Ht mice]. Gan No Rinsho.

[45] Park R L. (2000). Voodoo Medicine in a Scientific World.

[46] Watts G (2007). Let's pension off the "major breakthrough". BMJ.

